# Imaging Features of Superficial and Deep Fibromatoses in the Adult Population

**DOI:** 10.1155/2012/215810

**Published:** 2012-06-28

**Authors:** Eric A. Walker, Jonelle M. Petscavage, Pamela L. Brian, Chika Iloanusi Logie, Kenneth M. Montini, Mark D. Murphey

**Affiliations:** ^1^Department of Radiology, Penn State Milton S. Hershey Medical Center, Penn State College of Medicine, 500 University Drive, Hershey, PA 17033, USA; ^2^Department of Radiology and Nuclear Medicine, Uniformed Services University of the Health Sciences, 4301 Jones Bridge Road, Bethesda, MD 20814, USA; ^3^American Institute for Radiologic Pathology, 1010 Wayne Avenue, Suite 320, Silver Spring, MD 20910, USA; ^4^Department of Radiology, Walter Reed National Military Medical Center, 8901 Rockville Pike, Bethesda, MD 20889, USA

## Abstract

The fibromatoses are a group of benign fibroblastic proliferations that vary from benign to intermediate in biological behavior. This article will discuss imaging characteristics and patient demographics of the adult type superficial (fascial) and deep (musculoaponeurotic) fibromatoses. The imaging appearance of these lesions can be characteristic (particularly when using magnetic resonance imaging). Palmar fibromatosis demonstrates multiple nodular or band-like soft tissue masses arising from the proximal palmar aponeurosis and extending along the subcutaneous tissues of the finger in parallel to the flexor tendons. T1 and T2-weighted signal intensity can vary from low (higher collagen) to intermediate (higher cellularity), similar to the other fibromatoses. Plantar fibromatosis manifests as superficial lesions along the deep plantar aponeurosis, which typically blend with the adjacent plantar musculature. Linear tails of extension (“fascial tail sign”) along the aponeurosis are frequent. Extraabdominal and abdominal wall fibromatosis often appear as a heterogeneous lesion with low signal intensity bands on all pulse sequences and linear fascial extensions (“fascial tail” sign) with MR imaging. Mesenteric fibromatosis usually demonstrates a soft tissue density on CT with radiating strands projecting into the adjacent mesenteric fat. When imaging is combined with patient demographics, a diagnosis can frequently be obtained.

## 1. Introduction

The fibromatoses are a broad group of fibroblastic proliferations with a similar histologic appearance containing spindle-shaped myofibroblastic cells, dense deposits of intercellular collagen fibers, variable amounts of extracellular myxoid matrix, and compressed and elongated vessels [1]. They vary from benign to intermediate in biological behavior. Intermediately aggressive lesions (locally aggressive) are characterized by infiltrative growth and local recurrence but an inability to metastasize [2] (Table 1). This paper will discuss imaging characteristics and patient demographics of the adult type superficial (fascial) and deep (musculoaponeurotic) fibromatoses. The imaging appearance of these lesions can be characteristic (particularly when using magnetic resonance imaging). When imaging is combined with patient demographics, a diagnosis can frequently be obtained. Primarily pediatric fibrous lesions such as juvenile aponeurotic fibroma, infantile digital fibromatosis, infantile myofibromatosis, fibromatosis colli, and aggressive infantile fibromatosis are not included in this paper.

## 2. Superficial Fibromatoses

 The superficial (fascial) fibromatoses arise from fascia or aponeuroses at palmar, plantar, penile (Peyronie disease), and knuckle pad locations. Of the superficial fibromatoses, palmar fibromatosis is the most common followed by plantar fibromatosis [3, 4].

## 3. Palmar Fibromatosis

 Palmar fibromatosis (Dupuytren disease) is the most common of the superficial fibromatosis, affecting 1%-2% of the general population and approximately 4% of the United States population [5–7]. It was first described by Dupuytren at the Hôtel-Dieu in 1831 and thus is also referred to as Dupuytren disease or contracture [8]. Palmar fibromatosis is rare in Asian and African populations but frequent in the Northern European countries of Norway, Iceland, and Scotland, with prevalence rates between 30% and 39% [7, 9].

 The etiology of palmar fibromatosis is believed to be multifactorial, including components of trauma, microvascular injury, immunologic processes, and genetic factors.

 Patients are typically over 65 years of age and the process is rarely seen in children. Males are affected 3-4 times more often than females and the disease is more severe in men [10].

 Clinically, patients present with painless, subcutaneous nodules involving the palmar aspects of the fingers, usually the fourth and fifth digits [2, 11]. The nodules may progress over months or years to fibrous cords or bands which attach to and cause traction on the underlying flexor tendons of the fingers [3]. This results in the flexion contractures known as Dupuytren contractures. The process is bilateral in 40–60% of patients [12]. Coexisting conditions include plantar fibromatosis, Peyronie disease, knuckle pad fibromatosis, diabetes mellitus, epilepsy, alcoholism, manual labor with vibration exposure, smoking, hyperlipidemia, complex regional pain syndrome, and keloids [3, 6, 13].

 Surgical intervention remains the treatment of choice, typically a selective fasciotomy. The decision to undergo surgical excision is determined by both patient symptoms and the presence of flexion contracture greater than 20 degrees at the metacarpophalangeal (MCP) joint or greater than 30 degrees at the proximal interphalangeal (PIP) joint [14]. A simple surgical excision is associated with a high rate of local recurrence (30% to 40%), frequently within one year [4, 15].

**Figure 1:**
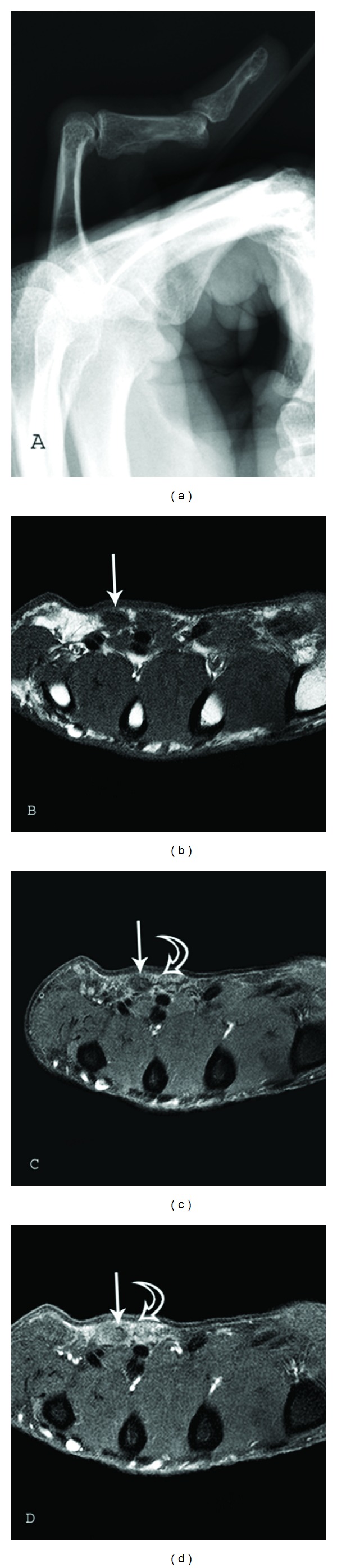
Palmar Fibromatosis. Lateral radiograph (a) of the right hand 5th finger in a 48-year-old man shows a flexion (Dupuytren) contracture of the proximal interphalangeal (PIP) joint. MR images of a 73-year-old man with pathologically proven palmar fibromatosis. (b) Axial T1-weighted (TR 500/TE 21) and (c) axial proton-density-weighted (TR 1500/TE 35) fat suppressed images of the hand at the level of the metacarpal bones show nodular areas of low-signal intensity in the volar subcutaneous fat (arrows) located superficial to the flexor tendons of the fourth and fifth fingers. Increased signal surrounds the nodules (curved arrow) on fluid sensitive sequence. (d) Axial T1-weighted (TR 500/TE 21) fat-suppressed image after administration of gadolinium contrast demonstrates moderate and diffuse enhancement (curved arrow) surrounding the nodules.

 Radiographs may be normal or demonstrate flexion (Dupuytren) contractures of the MCP and PIP joints (Figure 1(a)).

 On ultrasonography, the subcutaneous nodules of palmar fibromatosis are generally hypoechoic, hypervascular, and superficial to the flexor tendons [16, 17]. The extent of flexor tendon contracture can be assessed with dynamic ultrasonography.

 Computed Tomography (CT) demonstrates isoattenuated to slightly hyperattenuated nodular areas of subcutaneous thickening.

 MR imaging (MR) demonstrates multiple nodular or band-like soft tissue masses arising from the proximal palmar aponeurosis to the flexor tendons [18]. The nodules or bands of palmar fibromatosis usually measure between 2 and 10 mm in diameter. Signal characteristics have been shown to correlate with the cellularity of the lesions [19]. Low T1- and T2-weighted signal intensity are seen in hypocellular lesions composed of abundant dense collagen (Figures 1(b) and 1(c)). Cellular lesions have intermediate T1- and T2-weighted signal intensity with diffuse enhancement after the administration of gadolinium contrast (Figure 1(d)) [13]. This difference in MR signal intensity is important because the more cellular lesions have a higher local recurrence rate after excision [20]. Thus, these lesions could be managed by follow-up MR imaging to assess for change to lower signal intensity as an indication of maturation and decreased cellularity and to direct the optimal time for surgical intervention.

## 4. Plantar Fibromatosis

 Plantar fibromatosis (Ledderhose disease) (Morbus Ledderhose) occurs less frequently than the palmar lesion, with an incidence of 0.23% [4]. In our institutions, Ledderhose disease is more frequently imaged than Dupuytren disease. The etiology of plantar fibromatosis remains controversial, with prior trauma considered likely. Chromosomal variations have been seen in some lesions [3].

 Although the lesion can occur in children [21, 22], incidence increases with advancing age. In a large AFIP study (501 patients) 44% of patients were less than 30 years of age [1, 23]. Men are affected twice as often as females and lesions are bilateral in 20 to 50% of cases [22, 24].

 Patients present with one or more subcutaneous nodules, which most frequently arise in the medial aspect of the plantar arch (78%) and can extend to the skin or deep structures of the foot. Nodules may be multiple in 33% of cases [25]. Palmar fibromatosis is also present in 10% to 65% of patients with plantar fibromatosis. Other coexisting morbidities include diabetes mellitus, epilepsy, keloids, and alcoholism with liver disease [3, 21, 22, 26]. Most lesions are asymptomatic, only becoming symptomatic when the lesion invades adjacent structures such as neurovascular bundles, muscles, or tendons. Alternatively, some patients complain of aching pain after walking or standing for long periods of time. In contradistinction to palmar fibromatosis, plantar fibromatosis does not usually produce contraction deformities of the foot [3]. 

 The treatment of plantar fibromatosis is often conservative and consists of footwear modifications aimed at relieving symptoms [27]. Surgical resection is reserved for large lesions which cause significant disability and are refractory to nonoperative methods of management. Surgical treatment consisting of simple excision resulted in high rates of local recurrence (20%–40%), with the majority of lesions recurring within the first postoperative year [3].

 Radiographs are frequently normal in patients with plantar fibromatosis.

**Figure 2:**
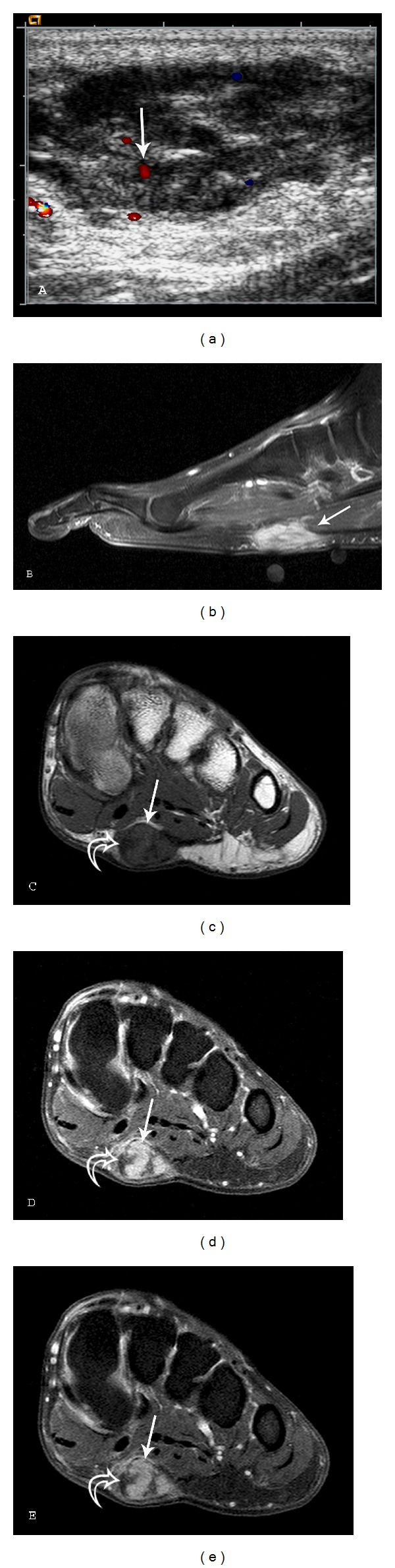
Plantar fibromatosis: a 54-year-old male who presents with left foot pain for one year. A longitudinal ultrasound color Doppler image (a) demonstrates a soft tissue mass with heterogeneous echotexture and internal color Doppler flow (arrow). (b) Sagittal T1-weighted (TR539.4/TE15) fat saturation postcontrast sequence demonstrates a fusiform, enhancing lesion with linear extension (fascial tail sign) along the plantar aponeurosis (arrow). Short axis MR images ((c)–(e)) demonstrate a well-defined mass (arrows) in the medial aspect of the plantar aponeurosis (c) Short axis T1-weighted (TR568/TE15) sequence reveals lesion signal intensity similar to skeletal muscle. There is heterogeneity with several foci of low signal (curved arrows) within the lesion. (d) Short axis T2-weighted (TR2693/TE60) with fat suppression reveals intermediate-to-high heterogeneous signal (arrow) and (e) T1-weighted (TR638.7/TE15) postcontrast fat saturation sequences demonstrate marked heterogeneous enhancement (arrow). Curved arrows indicate band-like areas of higher collagen content and low cellularity. Note the lower T1 and T2 signal intensity and lack of enhancement in these foci.

 Lesion evaluation is most commonly performed with ultrasound and MRI. Sonographic imaging demonstrates a well-defined (64%) or poorly defined (36%) fusiform mass in the soft tissues adjacent to the plantar aponeurosis (Figure 2(a)). Plantar fibroma may be heterogeneous and hypoechoic (76%) or isoechoic (24%) relative to the plantar fascia [3, 28]. Posterior acoustic enhancement (20%), cystic components, and intratumoral hypervascularity (8%) have also been described [28, 29].

 CT images demonstrate a nonspecific soft tissue mass in the characteristic location with attenuation equal or higher than skeletal muscle [3].

 MR imaging may demonstrate well-defined or ill-defined superficial lesions along the deep plantar aponeurosis, which typically blend with the adjacent plantar musculature. With its superior soft tissue contrast, MR is the best modality to determine infiltration of the lesion into the surrounding tissues and therefore it is most helpful for preoperative planning. Lesions typically show heterogeneous signal (92%), which is isointense to hypointense to skeletal muscle on T1W (100%) and T2W (78%) sequences. If the lesion has increased cellularity and less collagen, the T2 signal is increased (22%) [3]. The degree of enhancement has been reported as marked in approximately 60% and mild in 33% of cases [25]. Linear tails of extension (fascial tail sign) along the aponeurosis are frequent and best identified following intravenous contrast administration (Figure 2(b)) [3, 6].

## 5. Deep Fibromatoses

 The deep fibromatoses are fibroblastic proliferations that arise within the deep soft tissues and are traditionally divided into extraabdominal, abdominal wall, and intraabdominal types. They demonstrate infiltrative growth and local recurrence but do not metastasize. Their biological behavior may be considered intermediate due to frequent local recurrence. Involvement of adjacent vital structures may lead to patient demise, particularly with neck and chest wall lesions. Recurrent extremity lesions may eventually require amputation for local control [3, 6]. The World Health Organization (WHO) now groups these lesions together under the term deep or desmoid-type fibromatoses [2]. The term desmoid is derived from the Greek word desmos, meaning a band or tendon [30].

 The overall incidence of desmoid type fibromatosis is two to four individuals per million each year [31, 32]. Relative frequency of the individual subtypes of deep fibromatosis has been reported as abdominal wall (49%), extraabdominal (43%), and mesenteric (8%) [31]. Etiology is multifactorial with genetic, endocrine, and physical factors believed to play a role in pathogenesis. In the pediatric population there is an equal sex distribution and most lesions are extraabdominal. Patients from puberty to age 40 tend to be female and the abdominal wall is the most frequent site. After age 40, the sex distribution is again 1 : 1 with occurrence in abdominal wall and extraabdominal locations being approximately equal [33].

## 6. Extraabdominal Fibromatosis

 Synonyms for extraabdominal fibromatosis include musculoaponeurotic fibromatosis, extraabdominal desmoid, desmoid tumor, well-differentiated nonmetastasizing fibrosarcoma, and aggressive fibromatosis [1, 6].

 Extraabdominal fibromatosis is most common in patients between puberty and 40 years of age, with a peak incidence noted between the ages of 25 and 30 years. Less than 5% of patients are younger than 10 years of age [1]. Women are more commonly affected than men [34, 35].

**Figure 3:**
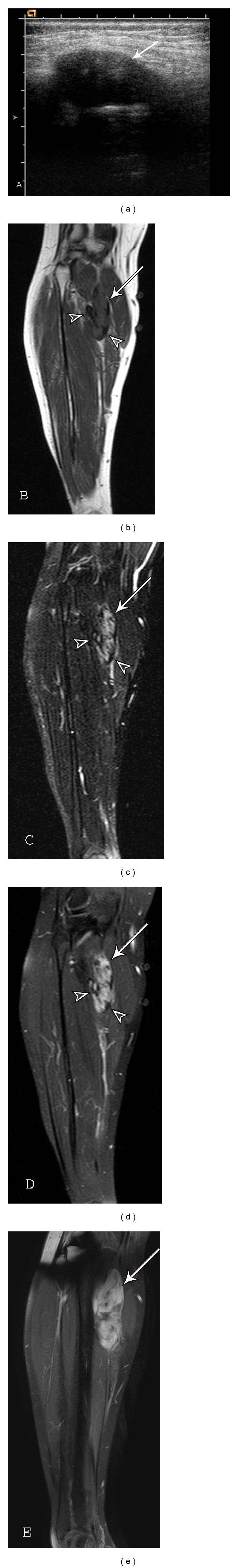
Extraabdominal fibromatosis of the popliteus and soleus muscles after pregnancy: a 27-year-old female with growth of a calf desmoid tumor noted during and after pregnancy. (a) Transverse sonography of the lesion (arrow) during a needle biopsy demonstrates a well-defined, heterogeneous hypoechoic mass. (b) T1-weighted (TR450/TE24) image reveals a heterogeneous lesion (arrow) measuring 6.6 centimeters (cm) × 2.9 cm in the coronal plane with signal similar to skeletal muscle. (c) Coronal T2- (TR3000/TE90) and (d) T1-weighted (TR450/TE24) postcontrast sequences with fat suppression shows a heterogeneous lesion (arrow) with central enhancement and significant band-like low signal component (arrowheads) predominantly at the periphery. (e) Coronal T1-weighted (TR484.913/TE7) postcontrast with fat saturation was obtained one year and nine months following the other MR images ((b)–(d)) and five months after partum. Both the size (9.8 cm × 3.3 cm in the coronal plane) of the desmoid tumor (arrow) and relative proportion of enhancing cellular tissue have increased under hormonal stimulation.

 While these lesions can occur almost anywhere in the body, they have a predilection for the upper torso including the upper arm (28%), chest wall/paraspinal (17%), and head/neck (10% to 23%). Other less common locations include the thigh (12%), knee (7%), buttock/hip (6%), and forearms (4%). Lesions in the head and neck often behave more aggressively and may surround the axillary vessels, trachea, and brachial plexus, limiting the extent of surgical resection [3, 6]. Extraabdominal desmoids are usually centered in an intermuscular region, although invasion of muscle is frequent. Endocrine factors may play a role in the development and growth of extraabdominal fibromatosis (Figure 3) [1].

**Figure 4:**
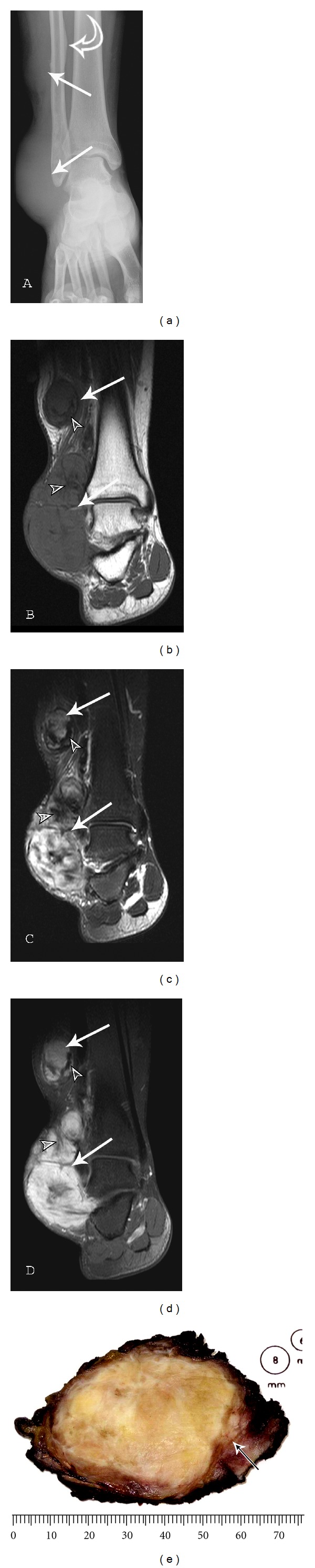
Multicentric extraabdominal fibromatosis of the right ankle: a 22-year-old male with ankle pain with prolonged standing. (a) AP radiograph of the right ankle demonstrates soft tissues masses (arrows) and mature periosteal reaction (curved arrow) of the fibula adjacent to the proximal lesion. (b) Coronal T1-weighted (TR420/TE10) sequence reveals predominantly intermediate signal lesions (arrows) of the subcutaneous tissues of the lateral ankle. (c) Coronal T2-weighted (TR4730.91/TE70) fat saturation image shows heterogeneous lesions (arrows) with moderate to marked enhancement on the (d) coronal T1-weighted (TR420/TE10) postcontrast fat suppression image. Low-signal-intensity bands (small arrowheads) of mature collagenized tissue within this desmoid tumor are best appreciated on the T2FS sequence. Photograph (e) of resected gross specimen demonstrates infiltrative borders (arrow) and a coarsely trabeculated surface.

 Although these tumors are usually solitary, synchronous multicentric lesions (Figure 4) are noted in 5% to 15% often in the same extremity (75% to 100%). Therefore a soft tissue mass in the extremity of a previously diagnosed desmoid tumor should be regarded as a second desmoid tumor until proven otherwise [30]. Multicentric fibromatosis can be associated with a skeletal dysplasia [36, 37].

 Extraabdominal fibromatoses typically present as a slow growing, painless mass which can limit range of motion of a nearby joint and invade adjacent neurovascular structures. Decreased range of motion, neurologic symptoms, and pain are reported but are unusual at presentation. Lesions are typically between 5 and 10 cm in size [6]. Extraabdominal fibromatoses have a tendency to grow along fascial planes and can extend a great distance from the predominant mass.

**Figure 5:**
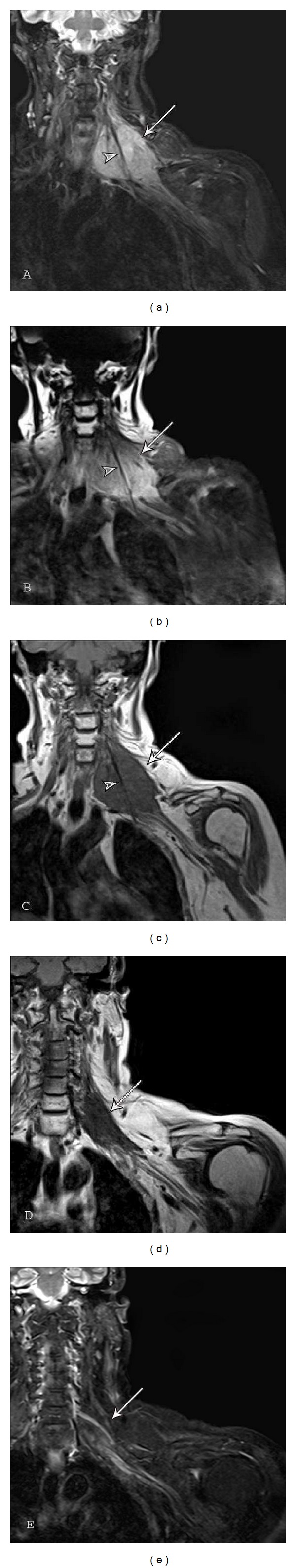
Extraabdominal fibromatosis of the left brachial plexus: a 64-year-old female presented with left shoulder and upper arm pain. The lesion was not amenable to surgical resection because of the intimate relationship with neurovascular structures of the left brachial plexus. (a) Coronal T2-weighted (TR4130/TE30) fat saturation and (b) coronal T1-weighted (TR576/TE11) postcontrast fat suppression sequences reveal a heterogeneous intermediate-to-high signal lesion with moderate and diffuse enhancement (arrows). Note the low-intensity band (arrowheads) corresponding to an acellular, collagen rich area interspersed between the highly vascularized fascicles of spindle cells. (c) Coronal T1-weighted (TR560/TE11) image obtained at presentation is compared to (d) coronal T1-weighted (TR572/TE14) and (e) STIR (TR5560/TE34) images obtained two years and four months after the previous images and status after completing radiotherapy (50.4 gray in 28 fractions). The lesion (arrows) reveals decrease in size and lower T1 and STIR signal indicating mature collagenized tissue after treatment.

 The treatment of extraabdominal desmoid is usually a wide-local excision as it has a high tendency to locally recur. Traditionally, positive surgical tumor margins upon resection have been reported to be associated with a higher local recurrence rate. However, more recent studies have suggested that positive versus negative microscopic margins do not make a difference in the overall local recurrence rate [38, 39]. Adjuvant radiation therapy following surgery has been shown to decrease the local recurrence rate versus surgery alone. In fact various studies have suggested that radiation therapy alone (Figure 5) in inoperable cases achieves near equivalent local control compared to surgery [39]. Additional therapies with reported positive results include radiofrequency ablation and chemotherapy agents such as Sorafenib (a multikinase inhibitor) and Imatinib (a protein-tyrosine kinase inhibitor) [39, 40]. Other modes of therapy include prostaglandin inhibitors and antiestrogen medications [41–43]. Although the lesion is not malignant, involvement of adjacent vital structures may lead to patient demise, particularly in lesions of the head/neck or chest wall [6].

 The rate of local recurrence varies from 19% to 77% (average 40%) and is usually within two years of resection [1, 2, 6].

**Radiographs**

**Figure 6:**
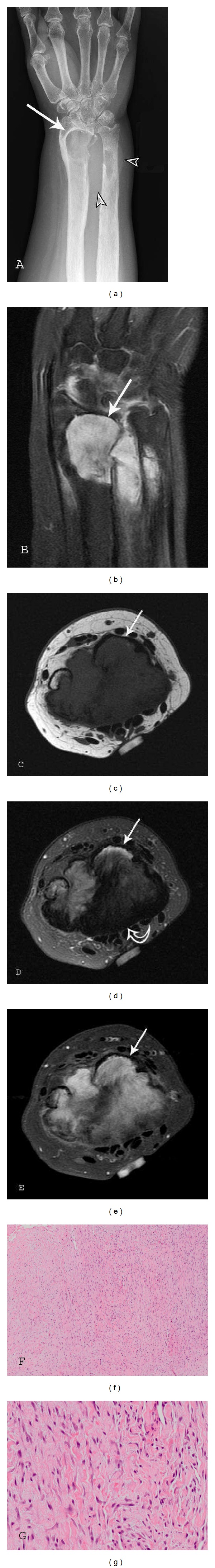
Extraabdominal fibromatosis of the right forearm with osseous involvement: a 47-year-old female with recurrent right distal forearm desmoid. (a) AP radiograph and (b) coronal T1-weighted (TR452/TE6.24) fat saturation (FS) postcontrast image demonstrate a distal forearm soft tissue mass (arrows) with involvement of the distal radius and ulna. Note the soft tissue density (arrowheads) on the radiograph. Axial (c) T1-weighted (TR428/TE9.5), (d) T2FS (TR3263/TE68.3), and (e) T1FS postcontrast sequences reveal a volar soft tissue mass deep to the flexor tendons with deep invasion and marrow replacement of the distal radius (arrows) and ulna. Low-signal, predominantly collagenous component (curved arrow) is best appreciated on the T2 fat suppression image. Photomicrographs (f) low- and (g) high-power hematoxylin-eosin (H-E) stain reveal spindled or stellate cells with bland nuclear features in a background of thick collagenous bands.

Radiographs usually appear normal, although there may be signs of a soft tissue mass such as the failure to visualize the radiolucent cleavage plane between the soft tissue and bone, structural changes of the adjacent bone such as scalloping, or rarely soft tissue calcification or ossification may be seen [26]. Bone involvement (Figure 6) is noted in 6% to 37% of cases [44, 45] and is more common after multiple recurrences [37].

**Nuclear Medicine**

Scintigraphy with Technetium-99m pertechnetate (Tc-99 m) has been used as a tumor scanning agent for follow-up of extraabdominal fibromatosis. Focal tracer accumulation is noted on blood pool and delayed static images [45].

**Ultrasound**

Sonography of desmoid-type fibromatosis reveals a hypoechoic lesion [46–51]. The lesions are illdefined and welldefined in similar frequency [51]. Color Doppler evaluation is useful in demonstrating vascularity of these lesions [6, 52]. Vascularity is absent in 66% of cases [51]. Lesions may demonstrate prominent posterior acoustic shadowing [47, 48]. One author describes visualizing a “fascial tail” sign and staghorn pattern with the use of high frequency ultrasound probes [53]. The “fascial tail” sign denotes linear tumor extension along the fascial planes and is described further in the section on MR imaging. The staghorn configuration corresponds to tumor extension of extraabdominal fibromatosis between subcutaneous fatty lobules. In our institutions, we use ultrasound primarily to guide needle biopsy of these lesions.

**Computed Tomography (CT)**

On CT, extraabdominal fibromatosis appears as a nonspecific soft tissue mass. Unless outlined by fat, the margins of the lesion are poorly defined. The attenuation of these tumors is variable, and has been described as lower than, similar to, or higher than skeletal muscle. Lesions with a higher attenuation are noted to have significant collagen components. One author suggests that a high attenuation lesion which is hypoechoic on ultrasound is suggestive of a fibrous lesion [49]. The low attenuation lesion is the least common pattern and likely reflects a significant myxoid component [3]. These lesions are well vascularized with numerous thick-walled capillaries resulting in enhancement on contrast enhanced CT and MR [54–56].

**Magnetic Resonance Imaging (MR)**

MR is frequently the modality of choice to evaluate and stage extraabdominal fibromatosis. Extraabdominal desmoid is usually centered in an intermuscular location, often along the deep fascia; therefore, a thin rim of surrounding fat may be noted (split-fat sign). Lesions may be well defined (49% to 54%) or have irregular infiltrative margins (46% to 51%) (Figure 8). T1-weighted sequences most often demonstrate lesions with intermediate signal intensity (isointense to muscle). The lesions are frequently heterogeneous, likely reflecting various proportions and distribution of collagen, spindle cells, and mucopolysaccharides within the lesion [6]. Studies have shown that the signal intensity of desmoid tumors varies according to tumor cellularity, as lesions with high fibroblast content demonstrate higher T2 signal and less cellular lesions demonstrate a lower signal intensity [57].

Three histopathological stages of desmoid tumor have been described. In the first stage, lesions are more cellular (Figure 3(a)), with larger extracellular spaces and less areas of hyalinized collagen. In the second stage (Figures 3(b)–3(d)), lesions demonstrate increasing amounts of collagen in the central and peripheral regions of the tumor. In stage three, increased collagen content is appreciated, with decrease in lesion cellularity (Figure 12(c)) and water content [58, 59]. These changes are best appreciated using MR imaging. Each of the three stages demonstrates lower T1- and T2-weighted signal intensity than the previous stage with stage three lesions revealing signal characteristics approaching that of tendon.

**Figure 7:**
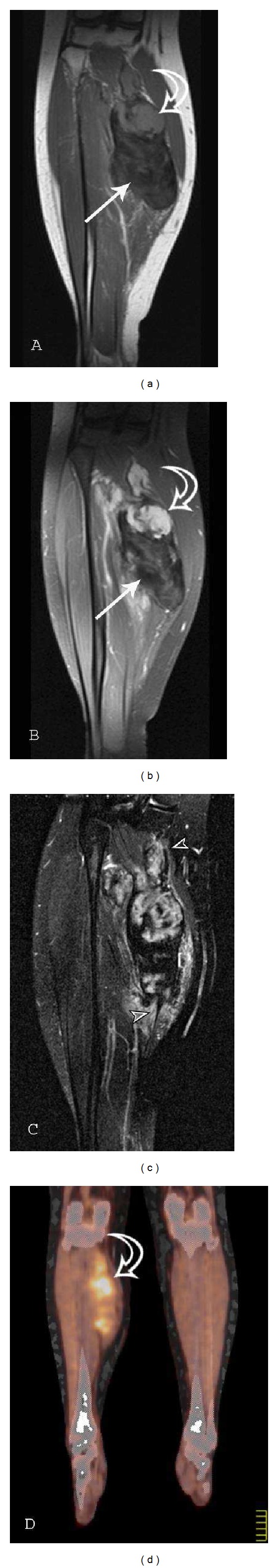
Extraabdominal fibromatosis of the medial soleus muscle: a 22-year-old female presents with a painless soft tissue mass of the right calf. (a) Coronal T1-weighted (TR550/TE12) and (b) coronal T1-weighted (TR552/TE12) postcontrast fat suppression sequences demonstrate a heterogeneous predominately low T1-weighted signal lesion of the medial soleus muscle (arrow). (c) Coronal T2-weighted (TR3000/TE70) image with fat saturation reveals the lesion growing along the fascia (fascial tail sign) (arrowheads) at the proximal and distal aspects of the lesion. (d) Coronal PET-CT fusion image reveals heterogeneous FDG uptake, which is the most common reported pattern of deep fibromatosis. Note the intermediate T1-weighted signal with marked enhancement corresponding to an area of high cellularity (curved arrows on (a) and (b)) at the proximal aspect of the lesion. This immature area demonstrates higher FDG uptake (curved arrow on (c)).

**Figure 8:**
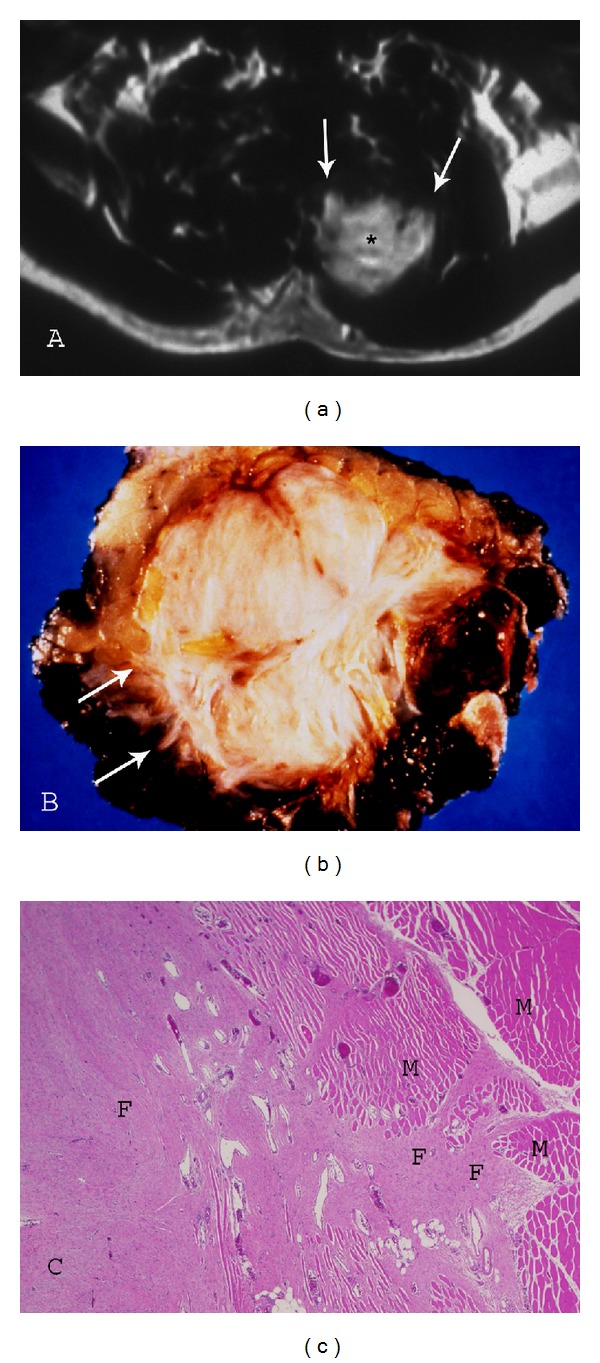
Paraspinal fibromatosis with infiltrative borders. (a) Axial postcontrast T1-weighted (TR500/TE20) sequence demonstrates fibromatosis of the paraspinal muscles with prominent enhancement (asterisk) and infiltrative margin (arrows). (b) Photograph of gross specimen reveals multiple collagenized bands and irregular, spiculated margin (arrows). (c) Photomicrograph (original magnification, *×*100; H-E stain) also illustrates the marginal invasion of muscle (M) by the collagenized fibromatosis lesion (F).

The morphology of these low-signal areas as prominent band-like regions (Figures 5(a)–5(c)) is more important to suggest the diagnosis [6]. These low-signal-intensity bands are common (62%–91% of cases) in desmoid type fibromatosis, compared with other neoplastic lesions, and are related to the collagenized, hypocellular bands seen at gross pathologic examination [3]. Following gadolinium administration, these collagenized bands demonstrate lack of enhancement. The low-intensity bands correspond to the acellular collagen rich areas which are interspersed between the highly vascularized fascicles of spindle cells. The administration of gadolinium causes these collagen bands to stand out in relation to the enhancing cellular areas of the neoplasm. Specificity of this pattern of enhancement has been reported in as high as 91% of cases [60]. Linear extension along fascial planes (fascial tail sign) (Figures 7(c) and 12(c)) is also a common manifestation of this lesion (83% of cases).

Signal intensity on long TR sequences may have an implication on tumor recurrence, with a higher recurrence rate in lesions with high T2 signal [6]. Lesions that respond to radiation therapy demonstrate progressive collagenization and show low-signal intensity on low TR images, and decrease in lesion size [6]. No significant enhancement is seen in approximately 10% of these lesions [61]. Tumor margins vary significantly, although they are usually well defined at presentation [62, 63]. Extension of the tumor along the fascia is very suggestive of extraabdominal desmoid. The fascial tail sign may also be seen with nodular fasciitis, abdominal wall fibromatosis and plantar fibromatosis.

In lesions undergoing radiation or drug therapy, MR surveillance has been used to assess response to treatment (Figure 5) with a positive response demonstrating a decrease in T2 signal, lesion enhancement and lesion size [3, 33].

Although not routinely performed in most institutions, it has been reported that diffusion-weighted imaging may help differentiate desmoid tumors from malignant soft tissue tumors, with fibromatosis demonstrating a higher mean apparent diffusion coefficient (ADC) than malignant soft tissue tumors [64].

Areas of low T2-weighted signal are not specific to fibromatosis and may be seen with other lesions. A differential diagnosis for soft tissue lesions with prominent areas of low-signal intensity on T1- and T2-weighted sequences includes desmoid type fibromatosis, densely calcified mass, pigmented villonodular synovitis (PVNS)/giant cell tumor of tendon sheath (GCTTS), elastofibroma, granular cell tumor, desmoplastic fibroblastoma, and malignant fibrous histiocytoma (MFH)/fibrosarcoma [20].

**Fluorodeoxyglucose Positron Emission Tomography (FDG PET)**

FDG PET has been utilized to evaluated decreased metabolic activity of all types of deep fibromatosis during chemotherapy [65, 66]. Desmoid tumors demonstrate maximal standardized uptake values (SUVmax) ranging from 3.4 to 5.4 in the literature. Heterogeneous FDG uptake was the most common reported pattern (Figure 7(d)).

## 7. Abdominal Wall Fibromatosis

 Abdominal wall fibromatosis is indistinguishable both grossly and histologically from extraabdominal fibromatosis and the relative frequency is similar. It is discussed separately because of its characteristic location and the tendency to occur in women of childbearing age (usually 20 to 30 years of age) during or more frequently within the first year following a pregnancy and in women who use oral contraceptives [21]. It is the most common soft-tissue neoplasm of the abdominal wall. Abdominal wall desmoids are solitary slow growing neoplasms that are recognized for their progressive, locally infiltrative, and aggressive behavior. Desmoid tumors involving the abdominal wall affect women in approximately 87% of cases, with 95% of these patients having had at least one child [1, 67]. These lesions often arises from the rectus abdominis or internal oblique muscles and their fascial coverings [1]. Abdominal wall desmoids tend to be smaller at detection than other types of deep fibromatosis (3 to 7 cm) likely because they become palpable at an earlier stage of lesion growth. A few cases have been reported in children of both genders. The majority of abdominal wall desmoids are solitary [6].

 Clinical presentation is typically palpation of a firm, slowly growing painless soft tissue mass. Endocrine factors are most highly implicated in this form of deep fibromatosis by the frequent occurrence of these tumors during or in the year following pregnancy. Estrogen receptors have been reported in 79% of these lesions [41] and they have been reported to regress at menopause [68]. Formation of these tumors in guinea pigs after prolonged estrogen exposure and prevention by administration of testosterone, progesterone, and desoxycorticosterone [69] as well as the estrogen inhibitors tamoxifen and raloxifen [70] has been reported. Similar to extraabdominal fibromatosis, abdominal wall fibromatosis may occur secondary to trauma. These lesions arise following a surgical procedure in 20% of cases, with 50% of these occurring within the first 4 postoperative years [71]. Abdominal wall desmoids have been reported arising at radical nephrectomy sites [72] and at the site of peritoneal dialysis catheter insertion [73]. Such lesions arising from scar tissue have been referred to as cicatricial fibromatosis. Abdominal wall fibromatoses may be associated with polyposis syndromes [74–76]. This is described in greater detail later in this paper.

 These lesions frequently recur locally. The rate of local recurrence is reported to be 15% to 30%, less frequent than extraabdominal desmoid (35% to 65%) [49, 77]. Similar to extraabdominal fibromatosis, wide local excision is the treatment of choice, and adjuvant radiation therapy may be needed for inoperable or recurrent lesions [50, 78].

**Imaging Features of Abdominal Desmoid**

**Figure 9:**
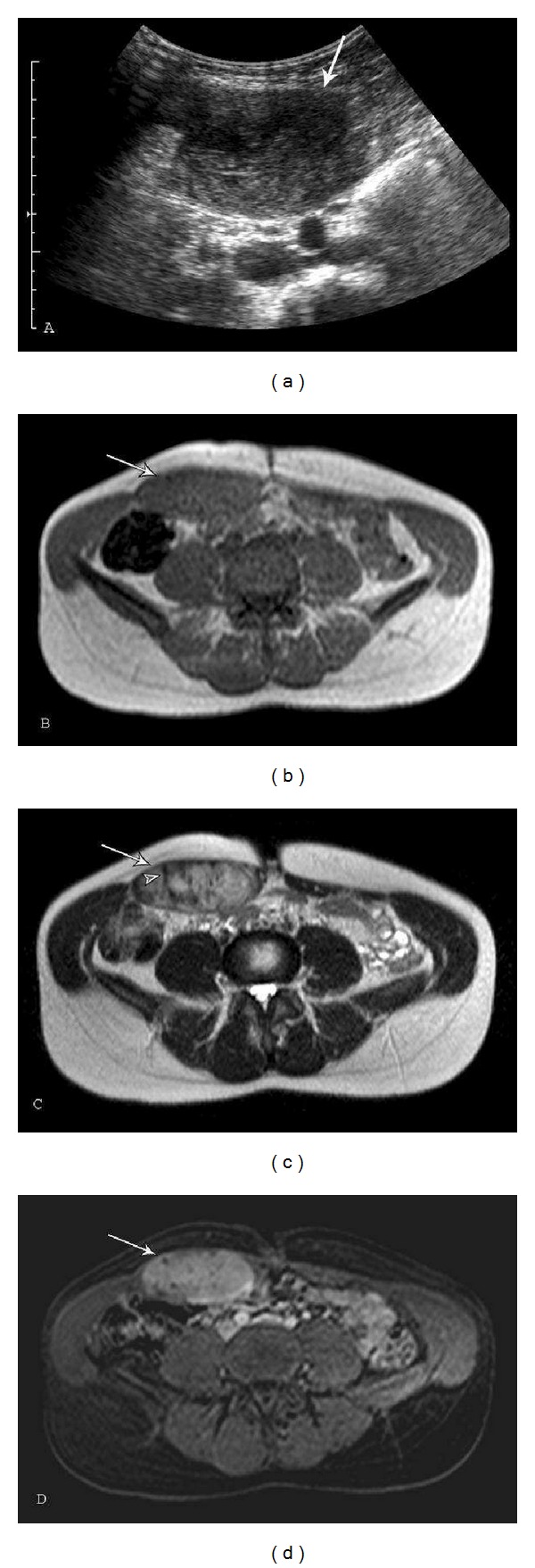
Abdominal fibromatosis of the right rectus abdominis muscle: a 35-year-old female with right rectus abdominis fibromatosis. The lesion continued to grow despite discontinuing oral contraceptive pills and taking ibuprofen. (a) Transverse ultrasound image demonstrates a well-defined, heterogeneous hypoechoic mass. (b) Axial T1- (TR148/TE4.6) and T2-weighted (TR441.2/TE100) sequences reveal a heterogeneous lesion (arrows) with T1 signal similar to skeletal muscle and intermediate to high T2 signal. Low-signal bands (arrowhead) are best evaluated on the (c) T2-weighted image. (d) Axial dynamic thrive (TR4.27/TE2.06) postcontrast fat suppression sequence reveals moderate and diffuse enhancement (arrow) of this lesion with high cellularity and scattered nonenhancing foci corresponding to the collagenized bands.

**Figure 10:**
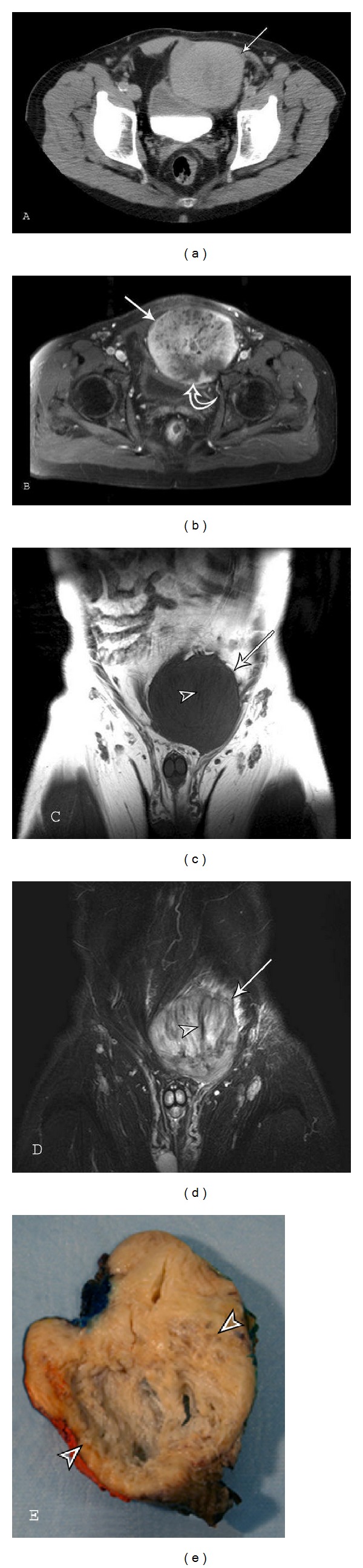
Abdominal fibromatosis: a 65-year-old male with a slowly growing painless suprapubic mass. (a) Axial CT and T1 (TE13/TR643) fat saturation image postcontrast reveal a well-defined soft-tissue mass (arrows) in the lower left rectus abdominis that displaces the bladder to the right. Lesion attenuation on CT is higher than skeletal muscle reflecting higher collagen component. An area of necrosis (curved arrow in (b)) is noted in the posterior aspect of the lesion. Lesion margins and heterogeneous enhancement are better appreciated on MR. (c) Coronal T1-weighted (TE11/TR427) and (d) STIR (TE78/TR4810) sequences show the lesion (arrows) with mildly ill-defined borders, mild peripheral edema, and band-like areas (arrowheads) of low signal within the lesion. (e) Photograph of the sectioned gross specimen, resected after radiation therapy, reveals a large area of central necrosis (arrowheads).

The radiologic features of abdominal wall desmoid, with all imaging modalities, are essentially identical to those of desmoid type fibromatosis in other locations. Involvement of the rectus abdominis muscle is most common (Figures 9 and 10). MR imaging is optimal for detecting the unusual manifestation of deep intraabdominal extension of tumor and guide resection. The fascial tail sign and low-signal-intensity bands (Figure 10(d)) also occur with abdominal wall desmoid and are valuable diagnostic clues [3].

## 8. Intraabdominal Fibromatosis

**Figure 11:**
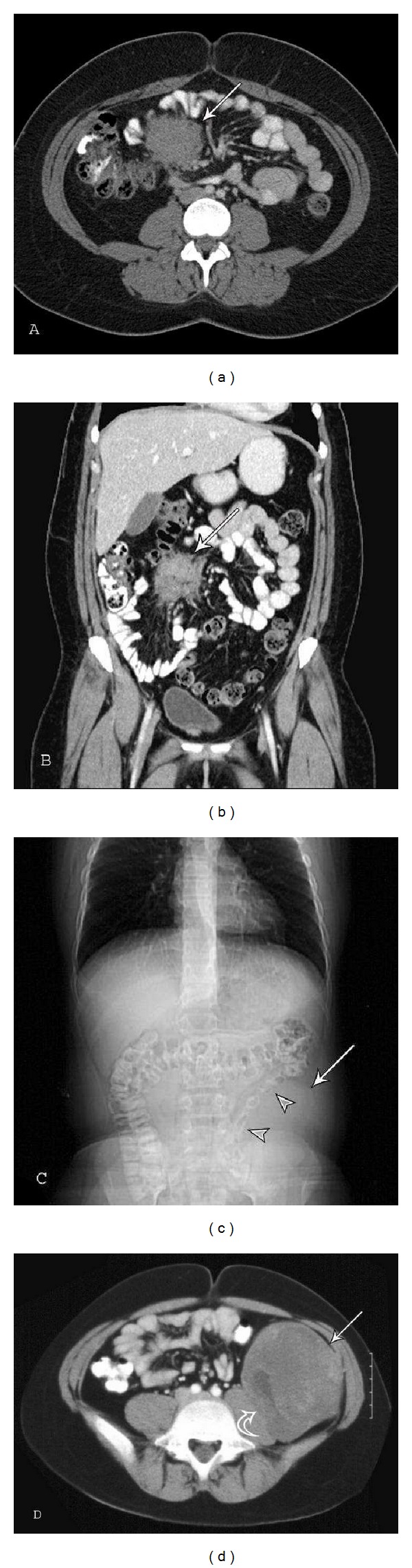
Intraabdominal fibromatosis. Mesenteric fibromatosis: a 29-year-old female with right-sided flank pain. (a) Axial and (b) coronal CT images demonstrate a mass (arrows) in the small bowel mesentery with irregular margins and attenuation similar to skeletal muscle. The mesenteric fat surrounds the lesion outlining the extent. Retroperitoneal fibromatosis: a 12-year-old male with a mass palpated in the left lower quadrant on routine physical exam. (c) CT topogram reveals an intraabdominal soft tissue mass (arrow) displacing the descending colon (arrowheads). (d) Axial CT shows a large retroperitoneal heterogeneous lesion (arrow) causing mass effect on the left psoas muscle (curved arrow) with scattered areas of mild-to-moderate enhancement.

 Intraabdominal fibromatosis (intraabdominal desmoid) is a rare group of closely related deep fibromatoses that occur in the pelvis, mesentery, and retroperitoneum (Figure 11). Despite their capacity to be locally aggressive, intraabdominal fibromatoses, like the other deep fibromatoses, do not metastasize [79]. The etiology remains unknown.

 Pelvic fibromatosis occurs in the iliac fossa and lower pelvis. It presents as a slowly growing palpable mass asymptomatic or causing slight pain. It is often mistaken for an ovarian neoplasm. The lesion occurs most frequently in women 20 to 35 years of age [1].

 The small bowel mesentery (Figures 11(a) and 11(b)) is the most common location for intraabdominal fibromatosis [80] and mesenteric fibromatosis is the most common primary tumor of the mesentery. It accounts for 8% of deep fibromatosis [1].

 Pelvic fibromatosis shows a female predilection, whereas mesenteric desmoids demonstrate a slight male predilection (55%) [81]. The age range of occurrence of mesenteric fibromatosis is 14 to 75 years of age, with an average age of 41 years [82]. They may become large before presentation, frequently 10 cm or more [1, 2].

 Intraabdominal fibromatosis demonstrates sporadic occurrence, but the incidence is increased in patients with familial adenomatous polyposis (FAP), trauma or hyperestrogenic states [1, 52, 83]. Intraabdominal fibromatosis is the type most commonly associated with Gardner syndrome [84]. Other hereditary disorders that are associated with mesenteric fibromatosis include familial infiltrative fibromatosis and hereditary desmoid disease [80].

 The presenting clinical signs and symptoms of mesenteric fibromatosis are often related to the small bowel. Patients may complain of abdominal pain or a palpable abdominal mass or come to clinical attention because of complications such as gastrointestinal bleeding, small bowel obstruction, fistula formation, bowel perforation, or hydronephrosis [5, 85, 86]. A differential diagnosis for mesenteric desmoid tumor would include lymphoma, metastatic deposits, carcinoid, sclerosing mesenteritis, gastrointestinal stromal tumor (GIST) and mesenteric lipodystrophy.

 Treatment options include surgical excision, hormone therapy, nonsteroidal anti-inflammatory drugs, and cytotoxic chemotherapy, although the optimal treatment regimen is controversial [87]. While surgical resection may be curative in sporadic cases, local recurrence is frequently encountered in patients with FAP [80]. There is a 23% overall recurrence rate (90% with Gardner syndrome and 12% without) [81].

**Radiographs**

Intraabdominal fibromatosis may result in local mass effect with displacement of adjacent bowel loops on radiographs (Figure 11(c)). Serosal changes can also be seen in the small bowel or colon mimicking mesenteric carcinoma or gastrointestinal stromal tumor (GIST) [79, 83].

**Ultrasound**

Mesenteric masses may be discovered incidentally when patients are being evaluated for complaints of abdominal pain or discomfort. The sonographic appearance of mesenteric fibromatosis is a solid, well-circumscribed mass of variable echotexture and homogeneity [72, 80, 83].

**Computed Tomography (CT)**

Mesenteric fibromatosis usually demonstrates a soft tissue density with radiating strands projecting into the adjacent mesenteric fat (Figures 11(a) and 11(b)) and attenuation directly related to the underlying histology. They can be hypoattenuating hyperattenuating or appear whorled because of the alternating collagenous and myxoid areas. Contrast enhancement is variable [80, 88]. CT is frequently the modality of choice for detection and follow-up of intraabdominal fibromatosis. The intraabdominal fat makes the lesion more conspicuous and bowel motion causes less artifact than with MR imaging. A recent paper suggests that MRI is at least equivalent (and possibly superior) to CT for the detection of intraabdominal desmoid tumors in FAP with the advantage of avoiding radiation to the patient [89].

**MRI**

Most intraabdominal lesions are low or intermediate signal intensity on T1-weighted images and have heterogeneous low-, intermediate- or high-signal intensity on T2-weighted images. The relative amount of hyperintensity on T2-weighted images reflects the degree of high cellularity areas and myxoid stroma within the lesion. The intravenous contrast enhancement pattern of mesenteric fibromatosis with MR is variable. Lesions that do not significantly enhance with iodinated contrast material on CT scans have been shown to enhance with intravenous gadolinium on MR imaging [80, 86]. In a study of mesenteric desmoids in FAP, 32% of mesenteric lesions demonstrated a whorled appearance, the majority of which demonstrated low T1- and T2-weighted signal [90]. Two papers concerning the MR characteristics of intraabdominal fibromatosis did not specifically mention a band-like pattern of low signal, but several of the figures appear to contain the characteristic band-like morphology [86, 90].

**Fluorodeoxyglucose Positron Emission Tomography (FDG PET)**

Mesenteric fibromatosis has been reported as a false positive during FDG PET for detection of metastatic disease [91, 92].

## 9. Deep Fibromatoses and Gardner Syndrome

**Figure 12:**
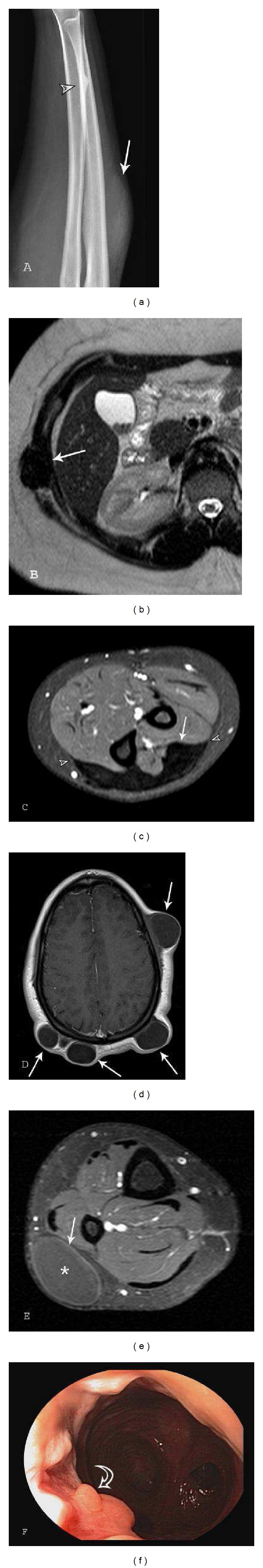
Imaging of gardner syndrome: a teenage female with Gardner syndrome. Images were obtained from age 13 to 16 years-of-age. (a) Lateral radiograph of the forearm demonstrates an osteoma (arrowhead) of the distal radial diaphysis. Also note the soft tissue mass (arrow) corresponding to extraabdominal desmoid imaged with MR in 11c. (b) Axial T2 (TR544.326/TE100) sequence through the upper abdomen demonstrates fibromatosis (arrow) involving the left intercostal muscles. (c) Axial T1 (TR539.09/TE15) fat saturation postcontrast of the upper forearm reveals a mature (collagenized) extraabdominal fibromatosis (arrow) along the dorsal superficial fascia with no significant enhancement. Note the fascial tail sign (arrowheads). (d) Axial T1 (TR491/TE11) postcontrast of the scalp and (e) axial T1 (TR667/TE10) fat suppression postcontrast of the calf reveal multiple epidermal inclusion cysts (arrows) of the subcutaneous tissues with mild peripheral enhancement. Follow-up endoscopy (f) of the patient status post colectomy for multiple tubular adenomas demonstrates development of an adenomatous polyp within the distal rectum.

 Gardner syndrome is a variant of familial adenomatous polyposis (FAP) containing osteomas, thyroid cancer, epidermoid cysts, fibromas, sebaceous cysts, and desmoid tumors in addition to the colorectal adenomatous polyps (Figure 12).

 Gardner syndrome is usually diagnosed in adults 25 to 30 years of age [1] and is more common in women. Approximately 2% of all desmoids are associated with FAP, and the incidence of desmoid tumors in FAP patients is approximately 850-fold greater than that of the general population [39]. The desmoid tumors in Gardner syndrome are postulated to form as a result of a mutation which affects the beta-catenin signaling pathway, which is commonly associated with familial adenomatosis polyposis syndrome [93].

 Patients with Gardner syndrome may demonstrate intraabdominal, abdominal wall, or musculoaponeurotic fibromatosis [94]. Of the subtypes of intraabdominal desmoid tumor, the mesenteric desmoids are more likely to be associated with Gardner syndrome and the pelvic and retroperitoneal desmoids are usually of the isolated form [6, 95]. Prior abdominal surgery (colectomy) is a risk factor for the development of mesenteric fibromatosis in patients with FAP [80, 83]. Recent case studies have reported these tumors forming in response to surgical instrumentation [96–98].

 Although similar in imaging characteristics to the isolated lesions, intraabdominal fibromatosis and other desmoid tumors associated with Gardner syndrome tend to be smaller and multiple and occur in a younger patient population. The smaller size and younger patient population may be secondary to more frequent imaging and follow-up examinations performed in patients with Gardner syndrome. Complications secondary to desmoid tumor are the most common cause of death in patients with FAP who have undergone prophylactic colectomy (30.6%) [99].

## 10. Conclusion

 In summary, the deep and superficial fibromatoses vary from low- (higher collagen) to-intermediate (higher cellularity) T1 and T2-weighted signal intensity depending on lesion cellularity. Palmar fibromatosis may result in flexion (Dupuytren) contracture and plantar fibromatosis typically involves the medial cord of the plantar fascia and blends with the adjacent plantar musculature. A heterogeneous lesion (well-defined or ill-defined) with nonenhancing low-signal-intensity bands on all pulse sequences and linear fascial extensions (fascial tail sign) are highly suggestive of abdominal wall or extraabdominal desmoid [6]. Mesenteric fibromatosis usually demonstrates a soft tissue density on CT with radiating strands projecting into the adjacent mesenteric fat. Patients with Gardner syndrome may demonstrate intraabdominal, abdominal wall or musculoaponeurotic fibromatosis. When imaging is combined with patient demographics, the diagnosis can frequently be suggested.

## Figures and Tables

**Table 1:** Characteristics of superficial and deep fibromatoses. The overall incidence of deep fibromatosis is two to four individuals per million each year.

	Superficial fibromatosis			Deep fibromatosis	
Characteristics	Palmar	Plantar	Abdominal wall	Intraabdominal (mesenteric)	Extraabdominal

Recurrence rate	30% to 40%	20% to 40%	15% to 30%	23% overall (90% Gardeners)	19% to 77%

Age	Often >65 years of age	3rd to 5th decade	20 to 30 years of age	Average 41 years of age	Peak 25 to 30-years-of-age

Sex	80% male	66% male	87% female	55% male	female predilection

Incidence	1% to 2% of population	0.23% of population	49% of deep fibromatoses	8% of deep fibromatoses	43% of deep fibromatoses

Association with Gardners	No	No	Yes	Yes with mesenteric subtype	Yes
